# Impact of the COVID-19 Pandemic on Trauma Evaluations for Violent Suicide Attempts and Self-Harm: A Retrospective Comparative Study

**DOI:** 10.7759/cureus.114082

**Published:** 2026-08-06

**Authors:** Austin Gould, Thaddaeus R Castaneda, James Ellis, Ella Potter, Jessica Kim, John J Leskovan, Amer A Afaneh

**Affiliations:** 1 General Surgery, Bon Secour Mercy Health - St. Vincent Medical Center, Toledo, USA; 2 General Surgery and Burn Surgery, The Oregon Clinic, Portland, USA; 3 Behavioral Health, Trinity Health Livonia Hospital, Livonia, USA; 4 Internal Medicine, Portsmouth Regional Hospital, Portsmouth, USA; 5 Trauma, Critical Care, and Acute Care Surgery, Bon Secour Mercy Health - St. Vincent Medical Center, Toledo, USA

**Keywords:** access to mental health services, covid-19, public health concern, self-inflicted injury, suicide risk

## Abstract

Introduction

The COVID-19 pandemic has exacerbated known risk factors for mental health deterioration, including economic instability, social isolation, and uncertainty, while also disrupting access to mental healthcare. Suicide remains a major public health concern in the United States, and self-inflicted injuries, although a minority of emergency department presentations, are associated with high morbidity and mortality.

Objective

This study examines whether the number of trauma evaluations related to violent suicide attempts or self-harm increased during the pandemic compared to pre-pandemic levels.

Materials and methods

We conducted a retrospective review of trauma registry data from one Level 1 and one Level 2 trauma center within Bon Secours Mercy Health, a regional hospital system. The pre-pandemic period extended from September 1, 2018, through February 29, 2020, and the pandemic period extended from March 1, 2020, through August 31, 2021. Adults aged 18 years or older who underwent trauma evaluation for a suicide attempt or self-harm were included. Patients younger than 18 years and those pronounced dead on arrival were excluded. The primary outcome was the number of trauma evaluations resulting from suicide attempts or self-harm. The secondary outcome was in-hospital mortality among patients who arrived alive and underwent trauma evaluation. Data were analyzed using two-tailed Wilcoxon two-sample tests, chi-square tests, and Fisher’s exact tests, as appropriate. Crude risk ratios with 95% confidence intervals were calculated for in-hospital mortality and ICU admission.

Results

A total of 147 trauma evaluations occurred among 129 unique patients during the study period. The pre-pandemic period included 76 evaluations among 61 unique patients, whereas the pandemic period included 71 evaluations among 68 unique patients, representing five fewer evaluations during the pandemic period. No patient underwent a qualifying trauma evaluation during both study periods. In-hospital mortality was 18.0% before the pandemic and 22.1% during the pandemic, corresponding to a crude risk ratio of 1.22 (95% CI, 0.61-2.46). ICU admission occurred in 42.6% of pre-pandemic patients and 50.0% of pandemic patients, corresponding to a crude risk ratio of 1.17 (95% CI, 0.81-1.71).

Conclusion

The pandemic period included slightly fewer trauma evaluations than the pre-pandemic period. No statistically significant differences were observed in the measured patient-level characteristics or in-hospital mortality between the study periods. Although the point estimates for in-hospital mortality and ICU admission rose slightly during the pandemic, the large confidence intervals reflected considerable uncertainty and prevented any conclusion that the periods were comparable. Repeat evaluations demonstrated continued utilization of trauma-system resources by a subset of patients. Larger multicenter studies are warranted to further evaluate temporal patterns in suicide-related trauma presentations.

## Introduction

The emergence of the novel coronavirus (SARS-CoV-2) pandemic in early 2020 created an unprecedented global public health crisis that extended beyond physical illness to include profound psychological and social consequences. The pandemic was associated with increased stress and psychological pressure among the general population, as reported in a 2020 study by Kawohl et al. [1]. In the United States, prevention measures, such as mandatory school closures, suspension of nonessential activities and productions, physical distancing, mandatory personal protective equipment, and quarantine, were implemented, all of which affected people's mental health, as mentioned in studies by Aquila I et al. and Dubey S et al. [2,3]. While these interventions were deemed necessary to mitigate viral transmission, they also contributed to increased social isolation, economic instability, disruption of mental health services, and heightened psychological distress across many populations. The constant flow of readily available information and reinforced messaging from online social networking services may have heightened mass hysteria and panic regarding COVID-19, as exemplified by studies by Dubey S et al. and Mamun MA et al. [3,4]. Many outpatient clinics were closed, demanding adaptations in the delivery of healthcare, especially mental health services [5]. These changes raised significant concerns regarding the potential impact of the pandemic on suicidal behavior and self-harm requiring acute medical care. 

Early literature raised concern that the pandemic could worsen anxiety, depression, substance use, and suicidal ideation, particularly among vulnerable populations [6-8]. The interplay between social isolation, financial hardship, reduced access to healthcare, and worsening psychiatric illness created concerns that suicide attempts and severe self-harm behaviors requiring hospitalization might increase during this period. However, published data evaluating changes in suicide-related trauma presentations have been inconsistent, with reports varying by geographic region, patient population, and study methodology. Trauma centers play a unique role in the care of patients who sustain violent suicide attempts or severe self-inflicted injuries. These patients require multidisciplinary management involving trauma surgery, emergency medicine, psychiatry, critical care, and rehabilitation services. In addition to the significant morbidity and mortality associated with these injuries, recurrent presentations and prolonged hospitalizations place significant demands on healthcare resources. Understanding trends in trauma center evaluations for violent suicide attempts and self-harm is therefore essential for resource allocation, trauma system planning, and development of integrated mental health interventions aimed at reducing recidivism and improving long-term outcomes. 

In 2018, suicide was the 10th leading cause of death in the United States, and an estimated 25 attempts occurred for every suicide death [9]. Although it accounts for a minority of ED presentations, self-inflicted injuries represent a high mortality and complication rate [10]. Prior mental health history, depression, polysubstance abuse, chronic pain, and alcohol-related factors have been associated with suicidal behavior and intentional injury [11,12]. The pandemic required adaptations in mental healthcare delivery, while psychiatric illness among trauma patients has been associated with poorer clinical outcomes [13-15]. Trauma assessment and management in patients with psychiatric disorders can be complicated by reduced adherence, self-neglect, psychiatric medications, and the need for more sedation. Patients who have attempted suicide or who have psychiatric disorders are likely at a higher risk for a complicated and prolonged hospital course with increased mortality after trauma [15].

Despite increasing recognition of the mental health consequences of the COVID-19 pandemic, relatively little is known about its impact on violent suicide attempts and self-harm requiring trauma center evaluation. Most existing studies have examined suicide mortality, emergency department visits, or psychiatric presentation, while comparatively few have focused specifically on patients presenting to Level 1 (L1) and Level 2 (L2) trauma centers with severe self-inflicted injuries. This represents an important knowledge gap, as this population differs significantly from patients with nonviolent suicide attempts in terms of injury severity, resource utilization, and multidisciplinary care requirements. The pandemic amplified known risk factors that contribute to poor mental health, and we aimed to determine whether this was evident at our trauma center [16-18]. The objective of our study was to compare trauma evaluations for violent suicide attempts and self-harm during the 18 months preceding the COVID-19 pandemic with those occurring during the first 18 months following its onset. We hypothesized that the pandemic would be associated with an increase in trauma center presentations related to violent self-harm and suicide attempts. By analyzing these trends, the study seeks to guide future trauma system preparedness and emphasize the need for coordinated, accessible mental health resources for patients with severe self-inflicted injuries during upcoming public health crises.

## Materials and methods

Study design

We conducted a retrospective observational cohort study using trauma registry data from one Level 1 and one Level 2 trauma center within Bon Secours Mercy Health, a regional hospital system. We compared trauma evaluations for violent suicide attempts or self-harm during the 18 months before the COVID-19 pandemic with those occurring during the first 18 months of the pandemic. The 18-month intervals were selected to assess the early impact of the pandemic while ensuring sufficient cases for statistical comparison. Data were analyzed for two periods: September 1, 2018, to February 29, 2020, covering the 18 months before the pandemic, and March 1, 2020, to August 31, 2021, covering the 18 months during the pandemic. The primary outcome was the number of trauma evaluations resulting from a violent suicide attempt or self-harm. The secondary outcome was in-hospital mortality among patients who arrived alive and underwent trauma evaluation.

Inclusion and exclusion criteria

Inclusion criteria consisted of all patients aged 18 years or older who presented to either trauma center during the study period and underwent evaluation by the trauma team for self-harm or a violent suicide attempt. Exclusion criteria included patients younger than 18 years of age and patients pronounced dead on arrival, as the latter did not undergo a complete trauma evaluation or inpatient clinical management.

Data collection

Data were collected via retrospective chart review of patients who presented to either hospital during the study period. Cases of violent suicide attempt or self-harm were identified based on patient self-report and/or EMS documentation. Variables collected included age, sex, mechanism of injury, Injury Severity Score (ISS), length of stay (LOS), ICU admission, intoxication status, mortality, and number of evaluations per patient. Intoxication status was based on documented blood ethanol and urine drug screen results. Analyses of intoxication status included only patients with available documented results.

Statistical analysis

Categorical variables and outcomes were summarized with frequency counts and percentages. Continuous or interval-scaled variables, such as age and ISS, were described using either mean and standard deviation or median and interquartile range, depending on the context. Age, ISS, and LOS were compared across study periods by combining data from both hospitals, and within each hospital during each period, using two-tailed Wilcoxon two-sample tests. Sex, intoxication status, ICU admission, and mortality were compared using chi-square or Fisher’s exact tests, as appropriate. The total number of trauma evaluations, including repeat evaluations, was reported separately from the number of unique patients. For comparisons of patient characteristics between study periods and trauma centers, each patient was counted once using the most recent evaluation within the applicable study period. Crude risk ratios with 95% confidence intervals were calculated for in-hospital mortality and ICU admission, with the pre-pandemic period serving as the reference group. The difference in the total number of trauma evaluations between study periods was reported descriptively because the counts included repeat evaluations and no population-based denominator was available. The comparison between the L1 and L2 trauma centers was exploratory and approached with caution, as no adjustments for multiple comparisons were made. Statistical significance was defined as two-tailed p < 0.05. Data analysis was conducted by an independent statistician using SAS version 9.4 (SAS Institute Inc., Cary, NC, USA). The materials and methods are summarized in Table 1.

**Table 1:** Study design, periods, centers, inclusion/exclusion criteria, outcomes, and variables collected Abbreviations: ISS, Injury Severity Score; LOS, length of stay; ICU, intensive care unit

Category	Details
Study design	Retrospective observational cohort study of trauma registry data from two hospitals (Level 1 and Level 2 trauma centers)
Study periods	Pre-Pandemic: September 1, 2018–February 29, 2020 (18 months)
	Pandemic: March 1, 2020–August 31, 2021 (18 months)
Centers included	Level 1 (L1), Level 2 (L2)
Inclusion criteria	Adults ≥18 years; evaluated by trauma service for violent suicide attempt or self-harm
Exclusion criteria	Pediatric patients; patients pronounced dead on arrival
Primary outcome	Number of trauma evaluations for violent suicide attempts or self-harm
Secondary outcome	In-hospital mortality among patients who arrived alive and underwent trauma evaluation
Variables collected	Age, sex, mechanism of injury, Injury Severity Score (ISS), length of stay (LOS), ICU admission, intoxication status, mortality, and number of evaluations per patient
Statistical methods	Wilcoxon two-sample test for continuous variables; chi-square or Fisher’s exact test for categorical variables; crude risk ratios with 95% confidence intervals for in-hospital mortality and ICU admission; descriptive comparison of total trauma evaluations

## Results

**Figure 1:**
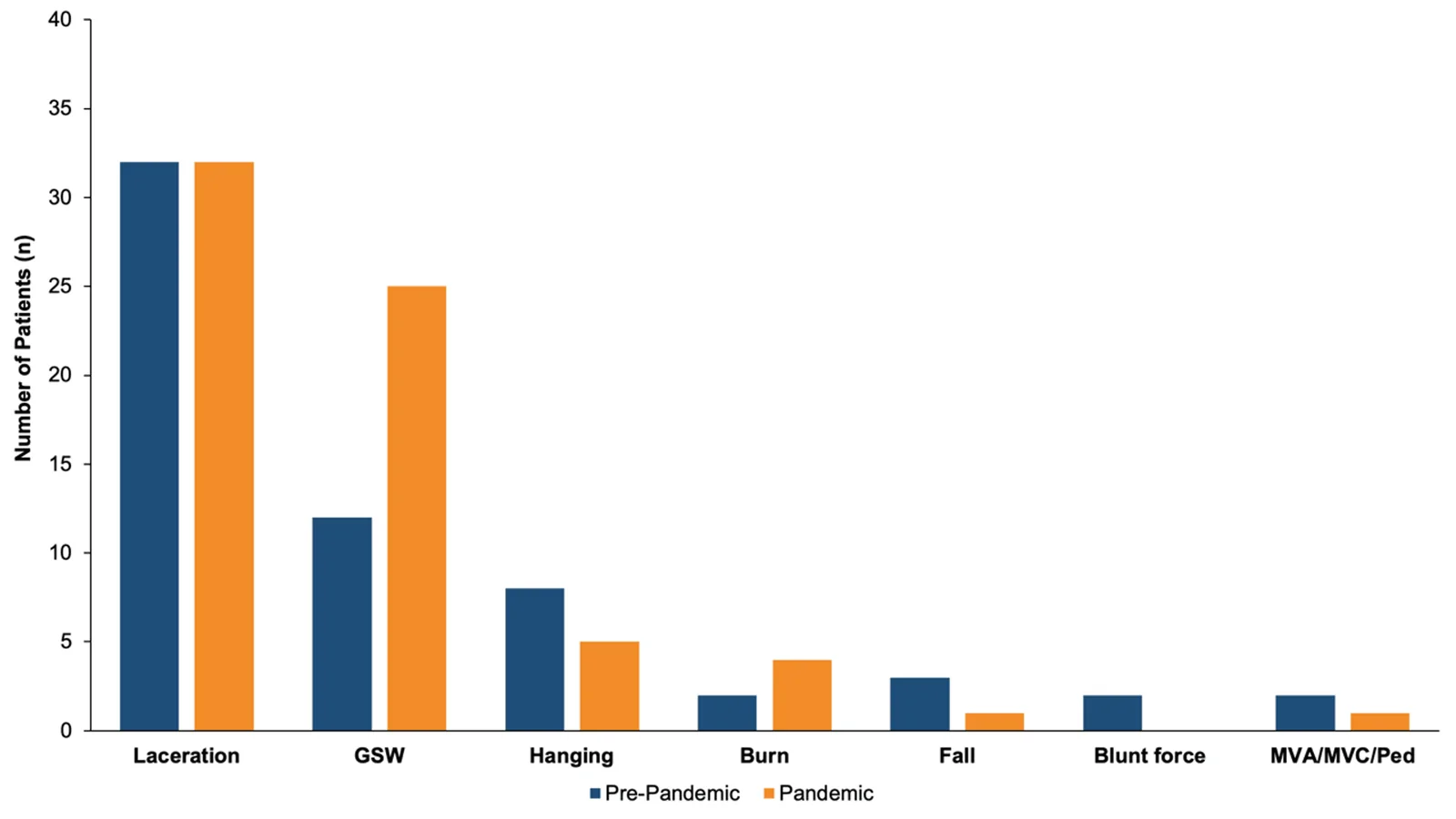
Mechanism of injury pre-pandemic versus pandemic (combined centers)

A total of 147 trauma evaluations occurred among 129 unique patients during the study period. The pre-pandemic period included 76 evaluations among 61 unique patients, whereas the pandemic period included 71 evaluations among 68 unique patients. No patient underwent a qualifying trauma evaluation during both study periods. Several patients underwent more than one evaluation within a single study period, as detailed in Table 2. Mechanisms of injury included laceration, gunshot wound, hanging, burn, fall, blunt force trauma, and motor vehicle collision or pedestrian versus vehicle injury. Laceration was the most common mechanism during both the pre-pandemic and pandemic study periods (Figure 1).

**Table 2:** Evaluations, unique patients, and recidivism by trauma center and study period Recidivism refers to repeated evaluations for violent suicide attempts or self-harm during the same study period. The date ranges shown represent the earliest and latest qualifying injuries observed at each center within the prespecified study periods.

Study period	Trauma center	Evaluations (n)	Unique patients (n)	Date range of injuries	Recidivism details
Pre-pandemic	Level 2 (L2)	32	20	9/12/2018–2/25/2020	One patient with 11 evaluations; two patients with two evaluations
Pre-pandemic	Level 1 (L1)	44	41	9/7/2018–2/29/2020	One patient with three evaluations; one patient with two evaluations
Pandemic	Level 2 (L2)	28	26	3/1/2020–7/28/2021	One patient with three evaluations
Pandemic	Level 1 (L1)	43	42	3/16/2020–8/25/2021	One patient with two evaluations

There were no statistically significant differences between the combined hospitals’ pre-pandemic and pandemic patients in age, sex, ISS, intoxication status, LOS, ICU admission, or in-hospital mortality (Table 3). The pandemic period included five fewer trauma evaluations than the pre-pandemic period, representing a 6.6% decrease. In-hospital mortality was 18.0% during the pre-pandemic period and 22.1% during the pandemic period, corresponding to a crude risk ratio of 1.22 (95% CI, 0.61-2.46). ICU admission occurred in 42.6% and 50.0% of patients, respectively, corresponding to a crude risk ratio of 1.17 (95% CI, 0.81-1.71).

**Table 3:** Comparison of patient characteristics pre-pandemic versus pandemic (combined centers) *Percentages reflect patients with available intoxication data.

Variable	Pre-pandemic (n = 61)	Pandemic (n = 68)	p-value
Age, mean (SD)	41 (16)	39 (15)	0.41
Female sex, n (%)	12 (20%)	14 (21%)	0.9
ISS, median (IQR)	9 (1–18)	9 (2–20)	0.56
Intoxication, n (%*)	13 (28%)	16 (34%)	0.55
LOS, median (IQR)	2 (1–4)	1 (1–5)	0.77
ICU admission, n (%)	26 (43%)	34 (50%)	0.4
In-hospital mortality, n (%)	11 (18%)	15 (22%)	0.57

Exploratory comparisons identified several differences between the L1 and L2 centers. During the pre-pandemic study period, patients at the L1 center had a longer LOS than patients at the L2 center (p = 0.03). ICU admission was more frequent at the L1 center than at the L2 center, although this difference did not reach statistical significance (51% versus 25%, p = 0.052; Table 4). During the pandemic study period, the proportion of patients with documented intoxication was higher at the L2 center than at the L1 center (p = 0.004; Table 5); however, this finding was limited by missing intoxication data. Patients evaluated at the L1 center also had a longer LOS (p = 0.01) and were more likely to require ICU admission (p < 0.001) than patients evaluated at the L2 center during the pandemic period (Table 5).

**Table 4:** Pre-pandemic comparison of patient characteristics by trauma center *Percentages reflect patients with available intoxication data. Statistical significance defined as p < 0.05.

Variable	Level 2 (n = 20)	Level 1 (n = 41)	p-value
Age, mean (SD)	41 (16)	41 (16)	0.87
Female sex, n (%)	2 (10%)	10 (24%)	0.18
ISS, median (IQR)	4 (1–17)	10 (4–21)	0.14
Intoxication, n (%*)	5 (42%)	8 (24%)	0.28
LOS, median (IQR)	1 (1–2)	2 (1–5)	0.03
ICU admission, n (%)	5 (25%)	21 (51%)	0.052
In-hospital mortality, n (%)	3 (15%)	8 (20%)	1

**Table 5:** Pandemic comparison of patient characteristics by trauma center *Percentages reflect patients with available intoxication data. Statistical significance defined as p < 0.05.

Variable	Level 2 (n = 26)	Level 1 (n = 42)	p-value
Age, mean (SD)	36 (12)	40 (17)	0.71
Female sex, n (%)	3 (12%)	11 (26%)	0.15
ISS, median (IQR)	9 (1–16)	10 (2–21)	0.55
Intoxication, n (%*)	9 (69%)	7 (21%)	0.004
LOS, median (IQR)	1 (1–1)	2 (1–7)	0.01
ICU admission, n (%)	6 (23%)	28 (67%)	<0.001
In-hospital mortality, n (%)	6 (23%)	9 (21%)	0.87

## Discussion

This study evaluated trauma center presentations for violent suicide attempts and self-harm during the 18 months preceding the COVID-19 pandemic compared with the first 18 months following its onset. Contrary to our initial hypothesis, no statistically significant differences were observed in the measured patient-level characteristics or in-hospital mortality between the two study periods. However, the relatively small sample size limits the precision of these findings and precludes concluding that the periods were equivalent. Although the point estimates for in-hospital mortality and ICU admission were modestly higher during the pandemic period, the confidence intervals were wide and included the null value, indicating substantial uncertainty regarding the direction and magnitude of any true differences. These results should not be interpreted as evidence that the pandemic had no effect on suicidal behavior. Rather, they indicate that, within our regional trauma system, the burden of severe self-inflicted injuries requiring trauma evaluation remained substantial throughout both periods.

Recent data, including a systematic review and meta-analysis conducted by Yan et al., demonstrated increases in suicidal ideation and suicide attempts, while suicide mortality remained stable [19]. Differences between those findings and the results of the present study may reflect the limited sample size and regional nature of our cohort. Pandemic management strategies differed significantly across cities and regions, as Jain et al. noted, potentially leading to discrepancies between local results and broader national or international data [20].

Our findings contribute to a growing body of literature evaluating the relationship between the COVID-19 pandemic and suicidal behavior, an area in which published results remain inconsistent. Several studies reported increases in suicidal ideation, depression, anxiety, and emergency department presentations for self-harm during the pandemic, while others demonstrated stable or declining suicide mortality rates. These discrepancies may reflect regional differences in healthcare access, public health restrictions, socioeconomic conditions, patient populations, and study methodology. Relatively few investigations have specifically examined patients requiring trauma center evaluation for violent suicide attempts and severe self-inflicted injuries. By focusing on this population, which frequently requires multidisciplinary management involving trauma surgery, critical care, and psychiatric services, this study addresses an important gap in the literature and provides insight into trauma system utilization during a major public health crisis.

A strength of this study is the use of real-world trauma registry data from L1 and L2 trauma centers, allowing assessment of patients treated across two institutions within a regional trauma system. The two equal 18-month observation periods provided a consistent descriptive framework for comparison. However, the study was not designed to adjust for seasonal or other temporal trends. The exploratory center-level comparisons and transparent reporting of nonsignificant findings provide additional context for future multicenter investigations.

We observed a clinically meaningful rate of recidivism, most notably one patient who accounted for 11 separate trauma evaluations during the pre-pandemic period. This finding underscores the disproportionate burden that a small subset of patients may place on trauma systems. Patients presenting after suicide attempts or severe self-harm remain at increased risk for subsequent suicide-related morbidity and mortality [15]. This population also requires substantial healthcare resources, as evidenced by ICU admission rates exceeding 50% at the L1 center during both study periods. The repeated evaluations observed in this study reinforce the importance of coordinated psychiatric assessment, trauma-informed care, discharge planning, and appropriate outpatient follow-up for patients at risk of recurrent self-harm.

At our L1 trauma institution, psychiatry is typically consulted when a patient is admitted to the trauma service following a violent suicide attempt or episode of self-harm. The institution has also introduced a Trauma Recovery Center (TRC) that assists trauma patients with mental health, financial, and social support in both inpatient and outpatient settings. Favorable outcomes have been reported with a hospital-based victim recovery program providing mental health and social support [21]. Although the present study did not evaluate the effectiveness of TRCs, the role of similar inpatient and outpatient support models for patients recovering from violent self-harm or suicide attempts warrants further study.

Several limitations should be considered when interpreting these findings. As a retrospective review, the study is subject to selection and referral bias and relies on existing medical records and trauma registry data. Inaccuracies in coding or classification of injury intent may have resulted in misclassification of eligible cases. The relatively small sample size of 129 unique patients and inclusion of only two regional trauma centers may have reduced statistical power to detect modest differences between study periods and limited generalizability to other healthcare systems. The absence of detailed psychiatric histories, prior suicide attempts, socioeconomic characteristics, and information regarding access to mental health services limited adjustment for important confounding variables that may influence suicidal behavior and healthcare utilization.

The cohort included only patients who survived long enough to undergo trauma evaluation and were captured within the participating trauma registries. Patients who presented to non-trauma centers, did not seek medical attention, or died before arriving at the hospital were not included, which may have affected the observed number and characteristics of presentations. Because the study was limited to trauma center evaluations, nonviolent suicide attempts managed by medical or psychiatric services were also not captured. Consequently, these findings reflect severe self-inflicted injuries requiring trauma evaluation and should not be generalized to overall patterns of suicidal behavior during the pandemic. In addition, intoxication analyses were limited to patients with available blood ethanol or urine drug screen results, and the center-level comparisons were exploratory and not adjusted for multiple comparisons.

Mental health remains a complex area influenced by clinical, social, economic, and behavioral factors. Trauma surgeons and multidisciplinary teams should remain cognizant that patients presenting after severe self-harm frequently require support and follow-up extending beyond treatment of their physical injuries [15,18]. Future multicenter prospective studies that incorporate detailed psychiatric, behavioral, and socioeconomic data, together with larger national datasets, will be essential to better define temporal trends in suicide-related trauma and to identify effective interventions to reduce recurrent self-harm and improve long-term patient outcomes.

## Conclusions

The pre-pandemic period included 76 trauma evaluations among 61 unique patients, whereas the pandemic period included 71 evaluations among 68 unique patients. No statistically significant differences were found in patient characteristics or in-hospital mortality. However, because of the limited sample size, we cannot determine if the two periods are equivalent. Repeated assessments, including 11 involving only a single patient, indicate continued use of trauma-system resources by some patients. These results highlight the clinical importance of multidisciplinary care, such as psychiatric assessment, coordinated discharge planning, and proper outpatient follow-up for individuals at risk of repeating self-harm. Larger multicenter studies with more detailed psychiatric, behavioral, and socioeconomic data are necessary to better understand the timing of suicide-related trauma and guide future prevention efforts and resource distribution.
